# The functional anatomy of central auditory processing

**DOI:** 10.1136/practneurol-2014-001073

**Published:** 2015-05-13

**Authors:** Thomas E Cope, David M Baguley, Timothy D Griffiths

**Affiliations:** 1 Department of Clinical Neuroscience, University of Cambridge, Herchel Smith Building for Brain and Mind Sciences, Cambridge, UK; 2 Department of Audiology, Addenbrooke's Hospital, Cambridge, UK; 3 Auditory Group, Institute of Neuroscience, The Medical School, University of Newcastle upon Tyne, Newcastle upon Tyne, UK

**Keywords:** Amusia, Auditory Agnosia, Central Auditory Pathway Disorders, Auditory Pathways

## Introduction

Disorders of audition stand at a crossroads between neurology, audiology, psychiatry and ENT. It is not uncommon for patients to ‘fall between the cracks’ of the referral pathway, and to become increasingly frustrated when specialties cannot decide who is responsible for them. The aim of this ‘How to understand it’ article is to demystify central auditory disorders for neurologists, and to show that they can be assessed like any other neurological symptom, based on a consideration of the anatomy and physiology of the auditory pathway. To illustrate this we describe the case history of a patient who had lost the ability to recognise and appreciate music who presented to an audiology clinician (DMB), who then sought neurology support (TEC, TDG). We elucidated the nature of the deficit in the analysis of sound patterns using psychophysics, but the deficits could have been predicted by a consideration of the anatomy and physiology of the auditory system.

## Case history

The patient was a 48-year-old, right-handed salesman who was a keen listener to popular music. He had no previous symptoms relating to environmental sound or speech perception.

**Figure 1:**
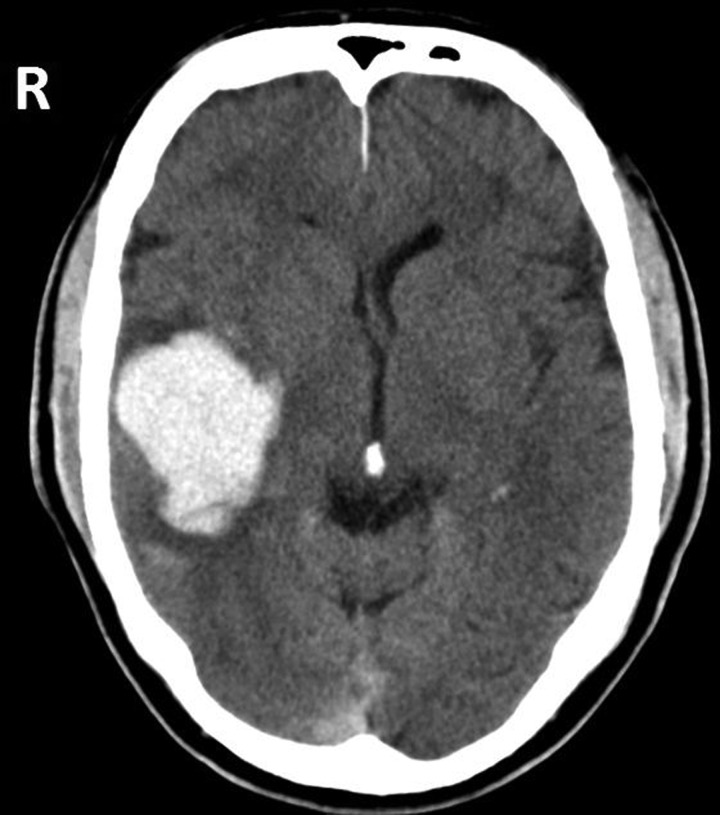
Unenhanced CT scan of head at the time of the first infarction. The area of haemorrhage involves almost the whole right temporal lobe, and extends into right parietal regions.

He suffered two low-speed road traffic accidents in a single journey on the way to work. The first was nose-to-tail, as he failed to see the car in front stop in traffic, and the second was nose-to-nose with a parked car. After the second incident, he pulled over and called an ambulance, as he felt ‘disorientated’. The ambulance crew found him to be confused and complaining of headache and nausea, so brought him to hospital. Initial physical examination showed no injuries but his blood pressure was 230/120 mm Hg. Neurological examination identified a left upper motor neurone facial palsy with preserved limb power, left-sided sensory and visual neglect, left-sided hyper-reflexia and left extensor plantar response. Immediate unenhanced CT of the head showed an extensive lobar haemorrhage, involving most of the right temporal lobe with some parietal lobe extension (figure 1). Digital subtraction angiography of brain was normal. He required 10 days in the high-dependency unit, primarily to control his elevated blood pressure, followed by rehabilitation on the stroke ward. He was discharged on day 28 with residual left hemianopia/neglect, and eventually made it back to work on reduced duties. Sometime later, 24-h ambulatory blood pressure monitoring was normal while taking amlodipine and ramipril. He described no new symptoms related to his hearing after the first episode.

**Figure 2:**
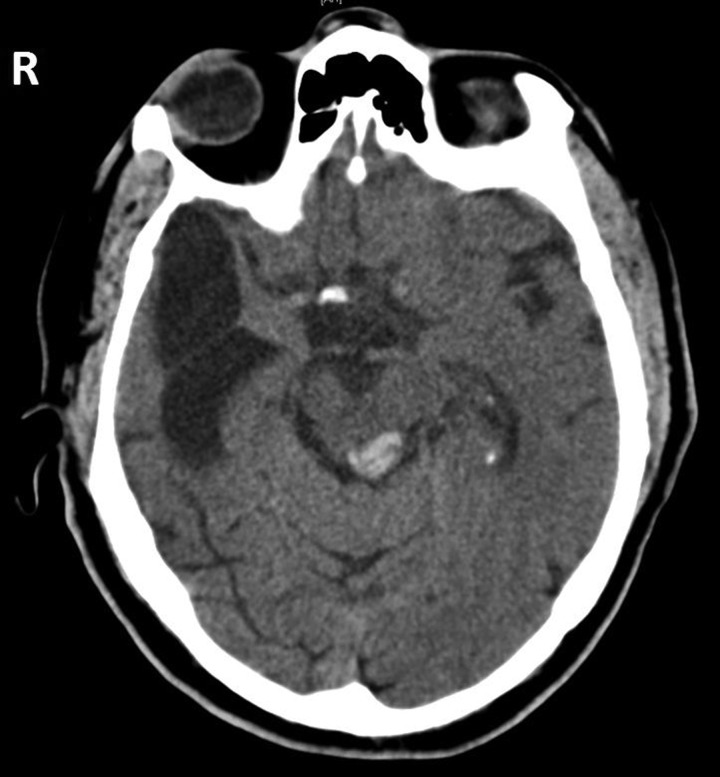
Unenhanced CT of head at the time of the second infarction. The area of haemorrhage is confined to the left internal colliculus. There is some secondary hydrocephalus (not shown), indicating obstruction of the cerebral aqueduct.

Four years later, he was woken from sleep by a tremendously loud noise, “like a spaceship landing”. He was disorientated, vomited and complained of headache and numbness of the right arm. He attended hospital, where it quickly became clear that he was completely deaf. Neurological examination was normal except for diplopia on left gaze, and his systolic blood pressure was 156/95 mm Hg. Over the next 12 h, his Glasgow Coma Scale score fell from 15 to 8 (E1, V2, M5) and his blood pressure rose to 214/76 mm Hg. Unenhanced CT of the head showed an isolated haemorrhage of the left inferior colliculus (figure 2) with obstruction of the cerebral aqueduct and consequent hydrocephalus. An intraventricular drain was inserted. The following day, he was fully conscious and orientated and able to communicate with whiteboard and pen. By discharge on day 14, he was aware of noises occurring but could not characterise them. He had no comprehension of speech, and suffered extremely loud and troublesome bilateral tonal tinnitus.

## Recovery of auditory function

**Figure 3:**
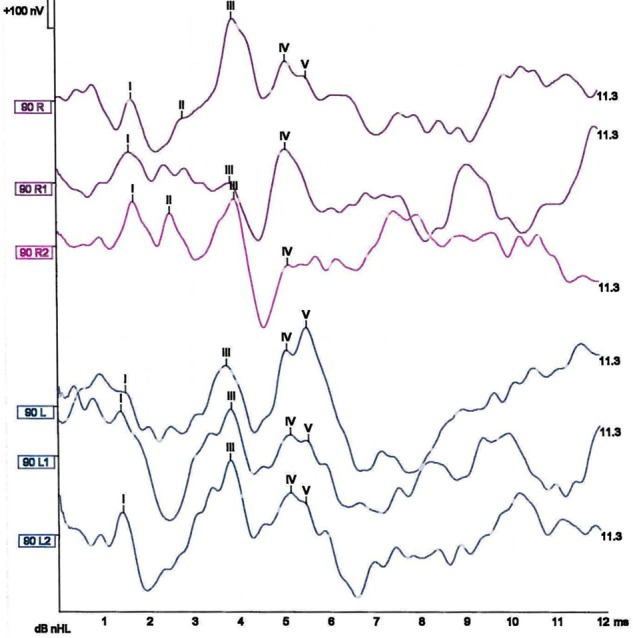
The patient's auditory brainstem responses to clicks taken 4 years after the second haemorrhage (unchanged from 4 years previously). The average responses to three sets of left-sided clicks are in blue at the bottom of the panel; these responses are normal. The average responses to three sets of right-sided clicks are in magenta at the top of the panel; these responses are abnormal, as there is no convincing presence of wave V, and the wave IV response is of small amplitude.

Over the next few months, his pure-tone audiometry (a test of hearing sensitivity) gradually improved until it was in the normal range for his age. Auditory brainstem-evoked responses (figure 3) were unchanged in the 4 years after the second event, showing a normal response to left-sided clicks, but absent waves IV and V (which are generated by the superior olivary complex and inferior colliculus in upper brainstem) to right-sided clicks.

Following audiology advice, during the 4 years after the second haemorrhage, he managed to retrain his understanding of speech by reading printed books while listening to the corresponding audiobook. This coincided with a gradual improvement in speech reception threshold (the minimum volume at which a patient can understand 50% of simple consonant-vowel-consonant words). More detailed testing of speech perception 4 years after the second event found a significantly impaired ability to distinguish minimal pairs of real words (eg, bear vs pear) but excellent performance distinguishing words from non-words (eg, bus vs mus).

In contrast to speech, 4 years after the second haemorrhage he still could not recognise music. Musical deficits were confirmed then by his performance on the Montreal battery of assessment of amusia.^1^ This assesses melodic and rhythmical structure systematically with a same–different task applied to pairs of novel melodies. The patient had a borderline performance on a test of contour (the pattern of pitch rises and falls) and was below cut-off for interval (degree of pitch change), scale (keeping in the same key), metre (march or waltz), rhythm and melodic memory (table 1).

**Table 1:** Scores on the Montreal battery of assessment of amusia

	Scale	Contour	Interval	Rhythm	Metre	Memory	Overall
Patient	17	22	15	17	17	15	103
Cut-off score	22	22	21	23	20	22	140
Amusic mean Peretz *et al*^1^	19	19	19	22	20	20	–

All tasks are scored out of 30 and are 2-alternative forced-choice, such that 15/30 represents chance performance. The first four tasks have catch (easy) trials to ensure concentration, and this patient answered all of these correctly.

Testing of uncued environmental sound recognition showed a small number of errors (table 2).

**Table 2:** Verbatim responses of the patient when asked to identify exemplars from a battery of sound effects

Sound effect	Response quotation	Correct?
General:		
Telephone	Bell, buzzer… telephone?	Yes
Door opening + closing	Fireworks, chainsaw… felling a tree?	
Cash register	No idea	
Crowd talking	Crowded room	Yes
Dentist's drill	The edge of a jungle	
Doorbell	Doorbell	Yes
Water pouring	Flushing toilet	Close
Pneumatic drill	Roadworks… jackhammer	Yes
Typewriter	Tap dancer	Close
Car engine + wipers	Factory floor	
Environment:		
Rain	Rain	Yes
Wind	Wind	Yes
Gentle waves	Thunderstorm	
Thunder	Louder thunderstorm	Yes
Fog (horn)	No idea	
Animals:		
Sheep	Lamb	Yes
Dog	Dog	Yes
Lion	No idea	
Birdsong	Birds	Yes
Cat	Cat	Yes
Cockerel crowing	Chicken… cock to be precise	Yes
Cow	Cow	Yes
Gorilla	Horse? Heavy breathing animal… could be any animal	Close
Horse	Not any inkling whatsoever	
Whale song	Elephant	

At the time of assessment, 4 years after the second haemorrhage, he therefore had deficits in auditory perception affecting speech, music and environmental sounds in the absence of deafness: an auditory agnosia.

## Auditory psychophysics

**Figure 4:**
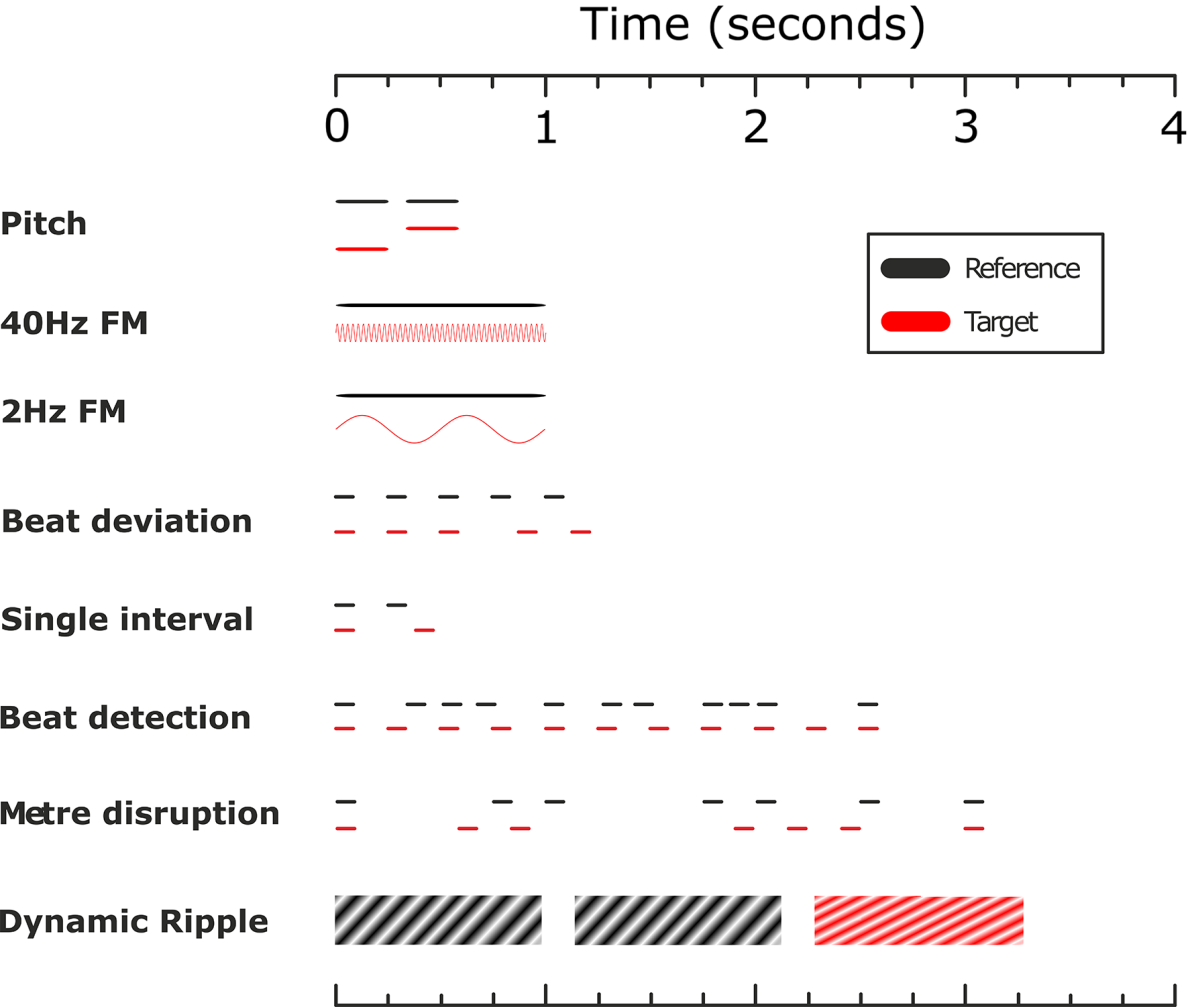
Cartoon illustrating the psychophysical tasks performed by the patient. The first six tasks followed a 2-alternative forced-choice paradigm, while the last two tasks (metre disruption and dynamic ripple discrimination) followed an ‘odd-one-out’ paradigm, again with two alternatives (and one template). Within each sequence the x-axis represents time, and the y-axis pitch height.

Complex percepts like speech and music rely on the analysis and integration of a variety of auditory cues or ‘building blocks’. Deficits in the analysis of particular sensory cues can produce deficits in the perception of complex sounds that contain that cue (eg, speech or music) in the absence of deafness. We suspected he had an ‘apperceptive’ agnosia. The presence of deficits that cross domains (here affecting music, speech and environmental sounds in descending order of severity) is common in apperceptive agnosia. To tease this apart further, the patient performed a range of psychophysical tasks that test the perception of different types of sound patterns and we compared this with group norms (demographic data for control groups in table 3). This establishes a profile of performance for the perception of different sound patterns, a bit like the neuropsychological profile measured in cognitive cases. The tasks used are illustrated in cartoon form in figure 4.

**Table 3:** Baseline characteristics of the patient, and the means of a group of 12 normal elderly controls from whom the auditory processing norms were established, and a group of 11 patients with peripheral neuropathies from whom the timing task norms were established

	Age	WTAR	MMSE	ACE-R	Att	Mem	Flu	Lan	Vis
Patient	56	42	30	99	18	25	14	26	16
Auditory processing controls	73	42	29	95	18	24	12	26	16
Timing task controls	60	42	29	94	18	24	12	25	16

ACE-R, Addenbrooke's Cognitive Examination; Revised edition, followed by subscores in attention, memory, fluency, language, visuospatial; MMSE, mini mental state examination; WTAR, Wechsler test of adult reading.

We first assessed pitch analysis. The perception of the pitch of individual notes is a complex process that depends critically on the fine timing information in sound at the level of milliseconds.^2^ His discrimination of pitch was strikingly abnormal (<0.1st centile), which is a sufficient low-level cause for the problems in melodic perception detected by the Montreal battery of assessment of amusia using more musical test stimuli.

The time structure of sound at the level of 10s of milliseconds is a critical feature relevant to speech and music perception. He was almost completely unable to detect frequency modulation at a rate of 40 Hz that is usually heard as a roughness (unmeasurably poor performance).

Tests of timing analysis in the 100s of milliseconds range found only mild deficits. Detection of a 2-Hz frequency modulation that is usually heard as a slow vibrato was borderline (2nd centile). Rhythm tests examining the 100s of milliseconds timing range showed borderline impairment in the detection of deviation from a regular beat (2nd centile), a high-level test of rhythm perception. In the same 100s of milliseconds range, he had normal discrimination of time intervals (54th centile), normal detection of the beat in a rhythmic sequence (17th centile) and normal metre detection (11th centile).

When asked to discriminate the spectral density of dynamic ripples (the ‘building blocks of speech’^3^)—essentially a higher level test of timbre perception in a difficult listening environment—he performed supranormally (>99th centile).

## Relating anatomy to time structure

**Figure 5:**
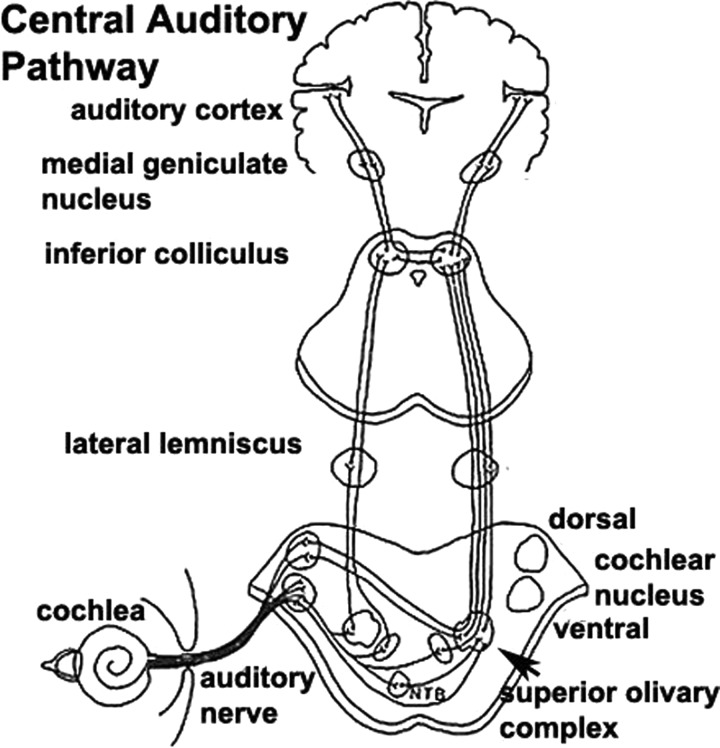
Schematic diagram of the auditory pathway from cochlea to primary auditory cortex. Adapted with permission from Hill.^42^ NTB, nucleus of the trapezoid body.

Taken together, the psychophysics demonstrate that the patient's most striking impairment is in the analysis of sounds with a time structure that evolves over milliseconds or 10s of milliseconds. To understand this, we must briefly consider the anatomy of the auditory pathway (figure 5). Inner hair cells in the cochlea transduce the mechanical energy of sound into electrical impulses in the auditory nerve which, for sounds below a few kilohertz in frequency, are phase-locked (ie, nerves fire at a fixed point in the cycle of a sound wave). After ipsilateral processing in either the dorsal or the ventral cochlear nucleus—and sometimes the nucleus of the trapezoid body—impulses are projected bilaterally, but with a contralateral dominance, to the superior olivary complex. The pathway travels up through the lateral lemniscus to inferior colliculus where there is a further partial decussation. There is then a further synapse in the medial geniculate nucleus before cortical processing in primary auditory cortex on Heschl’s gyrus of the medial temporal lobe. It is important to remember that, in contrast to the visual system, there is significant signal processing at each nucleus in the pathway.

As a result of this patient's initial haemorrhage, involving most of the right temporal lobe, we must assume that his cortical processing of auditory information is exclusively left sided. The second haemorrhage damaged the left internal colliculus, the highest level at which there is decussation of auditory tracts. This initially resulted in complete cortical deafness—as the left auditory cortex lost its meaningful input—and severe tinnitus, presumably as a result of disordered neuronal output from the internal colliculus. Over time, plasticity and perceptual retraining allowed him to make use of surviving ascending pathways to restore some useful hearing, but with permanent disruption of the synchronisation and temporal fidelity of these signals.

The cochlea represents the frequency spectrum of sounds in two ways, by the position on the basilar membrane with the largest amplitude of deflection,^4^ and by entraining neural firing to a fixed point in the cycle of a sound wave, so-called ‘phase locking’.^5^ Unlike in the visual system, where most postretinal processing occurs in the neocortex, auditory information is highly preprocessed by a series of brainstem nuclei before reaching the cortex. These nuclei are sensitive to temporal regularity, even when spectral information is absent.^6^ ^7^ Phase locking becomes progressively less precise as one ascends the auditory pathway, as this information is ‘distilled’ for further processing, freeing the neocortex from the necessity of encoding ‘pitch’ through regularity and allowing for the oscillatory activity common to other perceptual domains.^8^ Lesions to the brainstem, as in our patient, can therefore cause impairments in pitch discrimination.^9^ Because it is the first point of convergence of the auditory pathway, lesions of the brainstem also commonly lead to problems with integrating the inputs of both ears for spatial sound analysis.^10–12^

In our patient, his profound deficit for detecting acoustic features over timescales of milliseconds or 10s of milliseconds correctly localises the lesion to the auditory nerve or brainstem nuclei.^13^ ^14^ Deficits in temporal processing are unusual in brainstem lesions because of the incomplete decussation of the auditory pathway, which is about 70% crossed, meaning that lesions generally need to be bilateral to produce deficits. Bilateral brainstem lesions compatible with life are unusual but do occur in multiple sclerosis, in which there are well-described deficits in temporal sound analysis^15^

There is a general principle in the auditory system that the analysis of increasingly complex acoustic features over progressively longer time windows occurs at successive stages of the auditory pathway, and involves the recruitment of increasingly distributed networks.^16^ ^17^ Here we show abnormal analyses at timescales shorter than 10s of milliseconds due to a brainstem lesion. Difficulties with spectral flux, or the analysis of syllabic transitions over 10s of milliseconds localises to primary and then associated auditory cortices, as the time window lengthens,^18^ with a general trend for longer time windows to be processed in the non-dominant hemisphere.^19^ ^20^ Timing at the level of 100s of milliseconds and beyond recruits a distributed network^21^ involving the cerebellum,^22^ ^23^ the supplementary motor area^24^ and the basal ganglia.^25–27^

## Speech and music perception

Music is a fragile percept, and is less robust to degraded spectral structure than is speech perception.^28^ Amusia is therefore a relatively non-specific complaint in isolation, as it can result from deficits in a variety of auditory perceptual domains.^29^ This is illustrated by our patient, who performed well at tasks of timing at the level of 100s of milliseconds, the building blocks of rhythm, beat and metre, when time intervals were marked by tone pips in an otherwise silent background, but could not perform similar tasks in the Montreal battery of assessment of amusia when they were presented in a musical context.

By contrast, speech perception is a relatively redundant system. Humans can recognise speech when the auditory input is markedly degraded, especially after a period of training,^30^ and are aided by expectations, context and cross-modal cues such as lip-reading. This is illustrated by noise-vocoded speech,^31^ from which all temporal fine structure and the majority of spectral cues are removed, and which our patient could report to a normal level of accuracy.

When the difficulty with identifying and understanding music and speech is, as here, due to abnormal analysis of the structure of sounds, this represents an apperceptive auditory agnosia. In some other cases, the spectrotemporal analysis of sound is intact but there is abnormal association of the percept with meaning, this represents an associative auditory agnosia.

## Conclusion

In our experience, some neurologists dismiss auditory processing as being of ‘niche’ interest, and it is true that patients such as this are uncommon. Nonetheless, difficulties with auditory processing occur in a wide variety of ‘core’ neurological conditions. Multiple sclerosis is a common cause of brainstem lesions, and the efferent auditory pathway can be abnormal even with a normal MRI.^15^ Patients with aphasia resulting from stroke^32^ or neurodegeneration^33–35^ have significant difficulties with the processing of non-verbal sounds. Abnormal auditory sensations can occur in epilepsy, either as aura^36^ or after seizures,^37^ leading to a diagnosis of ‘focal epilepsy with auditory features’,^38^ which can be sporadic or related to mutation of the LGI1 ‘epitempin’ gene.^39^ Equally, emotionally valent sounds, and especially music, can precipitate seizures as part of ‘reflex epilepsies’.^40^ ^41^ In all cases, clinicians should attempt to link structure and function.

**Key points**

- Auditory agnosia is a deficit in identifying sounds that is not due to deafness: examples are word deafness (for speech sounds), phonagnosia (for voice), amusia (for music) and environmental sound agnosia.
- Auditory disorders can be assessed like any other neurological symptom, by relating structure to function.
- Auditory agnosia can be due to bilateral lesions of auditory cortex or from lesions affecting the ascending auditory pathway to cortex; it can evolve from cortical deafness.
- In this case the deficit was caused by complete destruction of the right auditory cortex and abnormal input to the left auditory cortex due to a highly localised lesion affecting the inferior colliculus: an obligatory relay to the left auditory cortex.
- It can be apperceptive (as here) due to abnormal analysis of the frequency and/or time structure of sounds; or associative, due to the abnormal association of sound percepts with meaning.
- Apperceptive forms often affect several domains (music and environmental sound here) due to a deficit in analysis of acoustic features relevant to multiple domains.
- In this case we showed a particular deficit in the analysis of rapidly changing sounds—which are analysed in the ascending pathway to cortex—as opposed to sounds that change slowly over time, which are relayed to cortex and first analysed there.
- The case can be understood in terms of the physiological principle that progressively longer time windows are analysed as signals ascend the auditory pathway, from brainstem to auditory cortex, and beyond to distributed processing.

**Further reading**

- Griffiths TD, Bamiou DE, Warren JD. Pathology of the auditory brain and its treatment. In: Palmer AR, Rees A, eds. *The Oxford handbook of auditory science: the auditory brain*. Oxford: Oxford University Press, 2010:509–43. ISBN-13: 978-0199233281.
- Cope TE, Sedley W, Griffiths TD. Disorders of audition. In: Toga AW, eds. *Brain mapping: an Encyclopedic Reference (chapter 138)*. Oxford: Elsevier Academic Press, 2015:1095–112. ISBN-13: 978-0123970251.
- Moore BCJ. *An introduction to the psychology of hearing*. 6th edn. Brill, 2012. ISBN-13: 978-1780520384.
